# ELIXYS - a fully automated, three-reactor high-pressure radiosynthesizer for development and routine production of diverse PET tracers

**DOI:** 10.1186/2191-219X-3-52

**Published:** 2013-07-12

**Authors:** Mark Lazari, Kevin M Quinn, Shane B Claggett, Jeffrey Collins, Gaurav J Shah, Henry E Herman, Brandon Maraglia, Michael E Phelps, Melissa D Moore, R Michael van Dam

**Affiliations:** 1 Department of Bioengineering, Henry Samueli School of Engineering, UCLA, Los Angeles, CA 90095, USA; 2 Department of Molecular and Medical Pharmacology, David Geffen School of Medicine, UCLA, 4323 CNSI, 570 Westwood Plaza, Building 114, Los Angeles, CA 90095, USA; 3 Crump Institute for Molecular Imaging, David Geffen School of Medicine, UCLA, Los Angeles, CA 90095, USA; 4 Sofie Biosciences, Inc., Culver City, CA 90230, USA

**Keywords:** Automated radiosynthesis, Fluorine-18, High-pressure synthesis, Multipot synthesis, Disposable cassette, FAC

## Abstract

**Background:**

Automated radiosynthesizers are vital for routine production of positron-emission tomography tracers to minimize radiation exposure to operators and to ensure reproducible synthesis yields. The recent trend in the synthesizer industry towards the use of disposable kits aims to simplify setup and operation for the user, but often introduces several limitations related to temperature and chemical compatibility, thus requiring reoptimization of protocols developed on non-cassette-based systems. Radiochemists would benefit from a single hybrid system that provides tremendous flexibility for development and optimization of reaction conditions while also providing a pathway to simple, cassette-based production of diverse tracers.

**Methods:**

We have designed, built, and tested an automated three-reactor radiosynthesizer (ELIXYS) to provide a flexible radiosynthesis platform suitable for both tracer development and routine production. The synthesizer is capable of performing high-pressure and high-temperature reactions by eliminating permanent tubing and valve connections to the reaction vessel. Each of the three movable reactors can seal against different locations on disposable cassettes to carry out different functions such as sealed reactions, evaporations, and reagent addition. A reagent and gas handling robot moves sealed reagent vials from storage locations in the cassette to addition positions and also dynamically provides vacuum and inert gas to ports on the cassette. The software integrates these automated features into chemistry unit operations (e.g., React, Evaporate, Add) to intuitively create synthesis protocols. 2-Deoxy-2-[^18^F]fluoro-5-methyl-β-l-arabinofuranosyluracil (l-[^18^F]FMAU) and 2-deoxy-2-[^18^F]fluoro-β-d-arabinofuranosylcytosine (d-[^18^F]FAC) were synthesized to validate the system.

**Results:**

l-[^18^F]FMAU and d-[^18^F]FAC were successfully synthesized in 165 and 170 min, respectively, with decay-corrected radiochemical yields of 46% ± 1% (*n* = 6) and 31% ± 5% (*n* = 6), respectively. The yield, repeatability, and synthesis time are comparable to, or better than, other reports. d-[^18^F]FAC produced by ELIXYS and another manually operated apparatus exhibited similar biodistribution in wild-type mice.

**Conclusion:**

The ELIXYS automated radiosynthesizer is capable of performing radiosyntheses requiring demanding conditions: up to three reaction vessels, high temperatures, high pressures, and sensitive reagents. Such flexibility facilitates tracer development and the ability to synthesize multiple tracers on the same system without customization or replumbing. The disposable cassette approach simplifies the transition from development to production.

## Background

Positron-emission tomography (PET) has opened the door to *in vivo* imaging for the purposes of non-invasive disease detection, cancer staging, and drug efficacy screening [1]. 2-[^18^F]fluoro-2-deoxy-d-glucose ([^18^F]FDG) is the most commonly utilized PET tracer due to its relative ease of production, manageable half-life, and ubiquitous application [2,3]. The increased demand for [^18^F]FDG has led to the development of many automated radiosynthesizers to lower its cost, enable its production at many different sites, and reduce the radiation exposure to the radiochemist [4,5].

Though automated synthesis of [^18^F]FDG is extremely valuable, there are many ^18^F-labeled PET tracers that await an automated synthesizer to streamline their production [6]. Some of these tracers require high pressures, complicated chemistry, and/or corrosive reagents that make automation difficult. For example, nucleoside analogs that have been used in imaging cell proliferation and reporter gene expression [7-9] and as possible screening agents for chemotherapy drug efficacy [10] often require high-temperature reactions in volatile solvents. Several attempts have been made to automate the syntheses of these tracers on commercially available radiosynthesizers. Often, these attempts have required modifications to the chemistry (e.g., use of alternative solvents or reduced temperatures) to reduce the pressures involved and avoid exceeding the limitations of the radiosynthesizers [8,11-16].

To overcome these synthesizer limitations, we previously developed a platform with movable components that seals the reaction vessel against an inert stopper during reactions to avoid exposure of tubing and valves to high pressures [17]. We further developed this system into a modular computer-controlled platform [18] and demonstrated the successful synthesis of 2-deoxy-2-[^18^F]fluoro-5-methyl-β-l-arabinofuranosyluracil (l-[^18^F]FMAU) and 2-deoxy-2-[^18^F]fluoro-β-d-arabinofuranosylcytosine (d-[^18^F]FAC). We describe here the integration of three of these modules into an automated synthesizer [19] and the addition of numerous improvements and features to increase the reliability and user-friendliness of the system. The ELIXYS three-reactor synthesizer is designed to use disposable cassettes for ease of setup and operation and to facilitate rapid transition from tracer development to routine production. The system also has an integrated, automated reagent handling robot to deliver sensitive reagents from sealed vials on demand. This paper describes in detail this radiosynthesizer and its characterization and validation via the synthesis of d-[^18^F]FAC and l-[^18^F]FMAU. The details of the software interface were published as a companion article [20].

## Methods

### Apparatus

The hardware and software of the ELIXYS radiosynthesizer were designed to be user-friendly and accommodate a wide variety of synthesis protocols by allowing the user to customize the reagents, reaction times, temperatures, and intermediate purifications in a manner that focuses on organizing automated synthesis steps into ‘unit operations.’ These chemistry operations serve as intuitive building blocks from which diverse syntheses can be constructed without the need for reconfiguring the instrument to perform different syntheses.

**Figure 1:**
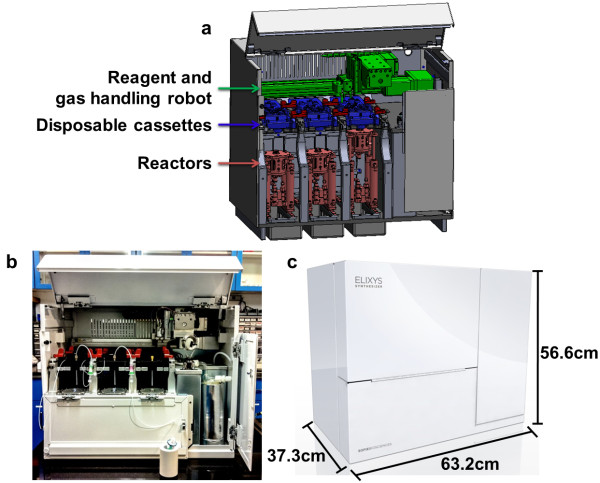
**ELIXYS synthesis module. (a)** Schematic, **(b)** photograph, and **(c)** enclosure with dimensions of the ELIXYS radiosynthesizer. The three reactors can be moved back and forth and raised to seal the vessel against various positions on the gasket at the bottom of the disposable cassettes to perform various unit operations. A reagent and gas handling robot manipulates reagent vials and supplies inert gas and vacuum for all three cassettes.

**Figure 2:**
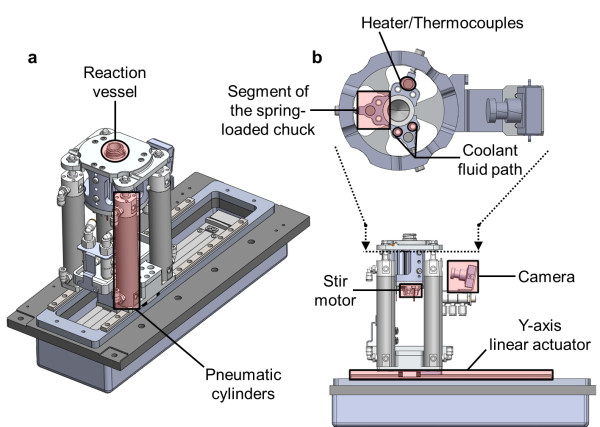
**Detailed view of a single reactor. (a)** 3D view of the reactor subassembly. **(b)** Side view (bottom) with a cross section through the spring-loaded chuck (top).

**Figure 3:**
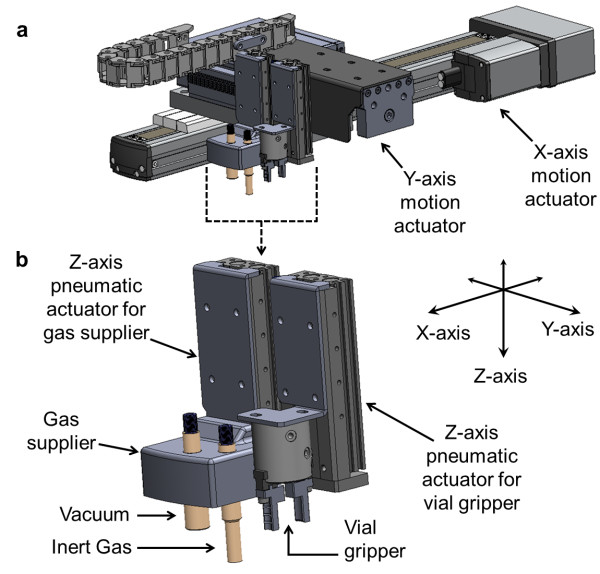
**The reagent and gas handling robot. (a)** Overview. **(b)** Close-up of the vial gripper and gas supplier head. The head moves in the *x*- and *y*-directions to access reagent vial positions and inert gas and vacuum ports on the three cassettes. The vial gripper and gas supplier can independently move in the *z*-direction.

**Figure 4:**
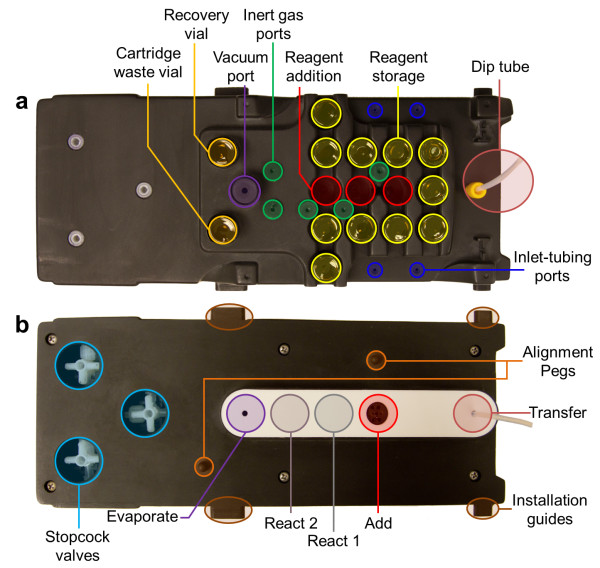
**Overview of the disposable cassette. (a)** The top view of the disposable cassette illustrates the locations for reagent addition and storage along with gas ports for the gas supplier to provide vacuum and inert gas. Tubing extends out of the inlet-tubing ports for radioisotope and external additions. **(b)** Bottom view. The reactor seals against various positions on a PTFE-coated silicone gasket underneath the cassette to perform various unit operations (Evaporate, React, Add, and Transfer). Each cassette contains three stopcock valves and has a dip tube for transferring products to purification cartridges, subsequent cassettes, or the HPLC injection valve.

The radiosynthesizer (Figure 1) has three key components working in concert: a set of three reactors (Figure 2), a reagent and gas handling robot (Figure 3), and disposable cassettes (Figure 4). The cassettes store reagents in sealed vials, act as the primary fluid path for both reagents and gas flow, and have a rubber gasket affixed to the bottom for sealing the top of glass reaction vessels. Cassettes accelerate setup, eliminate the need for cleaning, and facilitate a natural transition from tracer development to routine production. The reactor subassemblies provide temperature control of the reaction vessel and movement of the vessel to various positions beneath the cassette designed for evaporations, sealed reactions, reagent addition, and transfer of product. Once aligned at the proper position, the reactor is raised to seal the top of the vessel against the gasket on the underside of the cassette. The two-axis reagent and gas handling robot supplies inert gas (to drive fluid movement and assist with evaporations) and vacuum (to remove vapor during evaporations) to special interfaces on the top of the cassettes. A gripper mounted to the same robot manipulates reagent vials between storage and addition positions.

#### Reactor

A 5-mL glass V-vial (W986259NG, Wheaton, Millville, NJ, USA) is placed into the reactor and held within a three-segment spring-loaded ‘chuck’ (Figure 2). The segments press firmly against the vial to ensure excellent thermal contact and thus efficient heat exchange between the reactor and the glass vial. Each segment has one 100-W cartridge heater (CIR-1021-120V-100W-ST-A, Valin, San Jose, CA, USA) and a K-type thermocouple (HTTC72-K-116U-1.25-UNGR, Omega Engineering; Stamford, CT, USA) for individual feedback control of the reactor temperature. The maximum reactor temperature is 185°C, but we are investigating alternative types of fittings to enable an even higher operating temperature. Since we typically observed very similar temperature response in all three segments, the reactor temperature at any given moment is considered equal to the average of the three temperature readings from the thermocouples. Active liquid cooling is achieved by pumping room-temperature coolant (propylene/ethylene glycol and water mixture) through cooling channels in all three reactors in series using a liquid pump (8030-863-236, Steam Brite, San Antonio, TX, USA) and then through a radiator with three 140-mm fans (HX-CU1403V, Frozen CPU, East Rochester, NY, USA).

**Figure 5:**
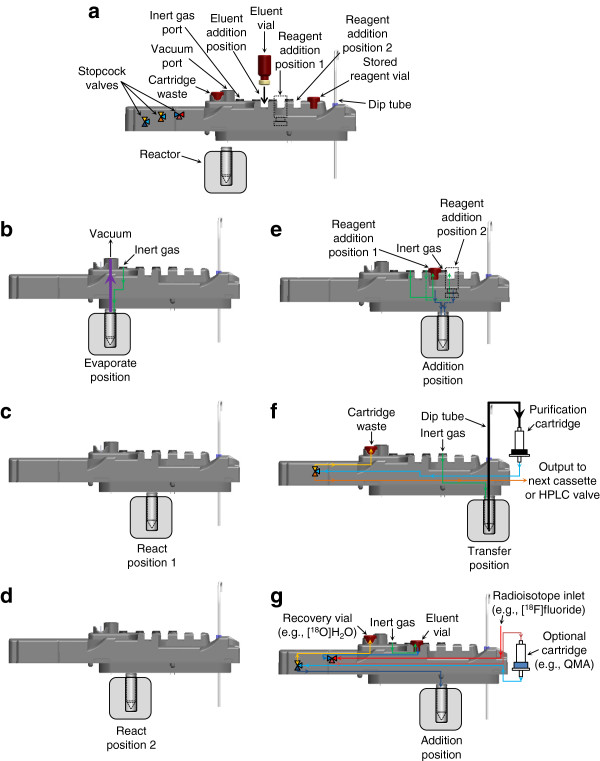
**Fluid diagram for each unit operation. (a)** Side profile schematic of the disposable cassette. The remaining figures show the schematic of cassette fluid paths for each unit operation. **(b)** Evaporate. Gas supplier provides vacuum and inert gas flow while the reactor is heated. **(c)** React1. First fully sealed reaction position. **(d)** React2. Second fully sealed reaction position. **(e)** Add. Vial gripper presses a reagent vial into one of two addition positions where two needles pierce the vial's septum; one needle allows inert gas flow from the gas supplier through the inert gas port, and the other needle allows the reagent to flow into the reaction vessel. **(f)** Transfer. Contents of one reaction vessel can be transferred to another cassette, the HPLC valve, or to a purification cartridge. **(g)** Radioisotope handling. [^18^F]fluoride trap and release can be done using two of the built in stopcock valves.

Each reactor is situated beneath a cassette and actuated back and forth among various positions (Figures 4 and 5). In each position, the reactor can be raised using pneumatic cylinders to seal the top of the reaction vessel against a portion of the gasket affixed to the bottom of the cassette. This movement allows for the reaction vessel and fluid path to be dynamically configured for different unit operations (Figure 5). For example, in one position, tubing is present to deliver reagents to the reactor; in another position, there is no tubing, allowing for a reaction under sealed conditions. Permanent tubing and valve connections to the reaction vessel are the root cause of the reaction pressure limitations of most synthesizers [17]. The ability to move the reaction vessel to a dedicated sealed reaction position eliminates these limitations and enables compatibility with higher pressures. To ensure reliable operation, the position of the reaction vessel is monitored via feedback from the linear actuator and the raised or lowered state is detected with Hall effect sensors (D-M9NWL, SMC Corporation, Noblesville, IN, USA).

We have mounted a camera (PC213XS, Super Circuits, Austin, TX, USA) behind the reactor, which is especially helpful during synthesis development to monitor liquid levels during evaporations, to observe visual cues for reaction progression, to confirm reagent additions and transfers, and for visual inspection of the eluate post purification. The final component of the reactor is a magnet mounted on a DC motor (803-313-5858, KALEJA Elektronik GmbH, Alfdorf, Germany) to rotate a removable magnetic stir bar inside the reaction vessel.

#### Reagent and gas handling robot

A pneumatically actuated vial gripper (Figure 3) that is affixed to a three-axis motion system grasps reagent vials and moves them from storage locations to addition locations (and vice versa) in the cassettes. The *x*- and *y*-axis motions are performed by a pair of linear actuators. The *z*-axis motion is accomplished with a pneumatic cylinder. Sealed reagent vials are installed upside-down in the cassettes (Figure 5).

A gas supplier, comprised of inert gas and vacuum supply ports, is mounted on a second pneumatic *z*-axis actuator affixed near the vial gripper (Figure 3). In the previous prototype to the ELIXYS [21], the cassettes each had multiple inputs on their bottom surface for inert gas and vacuum that mated with corresponding ports on the synthesizer. This system was prone to failure due to the large number of connections and the difficulty in maintaining reliable gas-tight seals during operation. With a single, movable gas supplying robot in ELIXYS, a large number of valves and seals are eliminated, increasing the reliability of the system. The gas supplier is lowered to engage respective vacuum and inert gas ports on top of the cassette (Figure 4). The cassettes contain a rubber gasket around each port to form a gas-tight seal with the port on the robot. The vacuum port is mounted at a height above the inert gas port and on a spring-loaded mechanism to ensure proper sealing of both ports when needed and to avoid collision of the vacuum portion of the gas supplier when vacuum is not needed.

The use of Hall effect sensors as feedback mechanisms on *z*-axis pneumatic actuators and the vial gripper enable detection of missing reagent vials and prevent *x*- and *y*-axis motions if the vial gripper and gas supplier are not in their raised, clearance positions. An in-line check valve (CI-5C, Bio Chem Fluidics, Boonton, NJ, USA) is installed on the inert gas line close to the delivery point to eliminate backflow of vapor. A cold trap (CG451501, Chemglass, Vineland, NJ, USA), cooled in a small Dewar (10-195A, Fisher Scientific, Pittsburg, PA, USA), typically with a mixture of dry ice and methanol, is installed in-line between the vacuum port and the integrated vacuum pump (VP0140-V1006-D2-0511, Medo USA Inc., Roselle, IL, USA) with a digital vacuum gauge (ZSE30-N7L, SMC Corporation).

#### Disposable cassettes

Cassettes (Figure 4) are designed to contain all disposable components and fluid paths, eliminating the need to clean or customize the apparatus between syntheses. These molded polyurethane cassettes contain stainless steel needles (Vita Needle, Needham, MA, USA), tubing, chemically inert three-way stopcock valves (EW-31200-80, Cole-Parmer, Vernon Hills, IL, USA), and a custom polytetrafluoroethylene (PTFE)-coated silicone gasket (Specialty Silicone Products, Inc., Ballston Spa, NY, USA, and Cannon Gasket, Upland, CA, USA) against which the reaction vessel is sealed. The fluid paths are shown in Figure 5. Preassembled cassettes slide along rails into the ELIXYS system which are then locked down by clamps. Accurate positioning is ensured by the mating of alignment features and by the engagement of stopcock valves with adapters affixed to rotary pneumatic actuators (CRB2BW20-180S, SMC Corporation).

Each cassette has 11 reagent vial storage positions that each house one 13-mm crimped septum-cap vial (with a maximum volume of 3 mL) in an inverted configuration. Three additional vial positions contain dual upward-pointing needles to pierce the septa for delivery of fluids from the reagent vials. One needle in each position is used for fluid delivery; the other connects to an inert gas port on top of the cassette, which allows pressurization of the vial by the gas supplier. In the two reagent addition positions, the fluid delivery needles output directly to the underside of the cassette where the reaction vessel is sealed for reagent addition. The fluid delivery needle in the third position (for eluent addition) is connected via internal tubing to a stopcock valve.

Two of the stopcock valves are used to reconfigure the internal fluid paths to perform cartridge trap and release, such as for the preparation of an incoming radioisotope, e.g., solvent exchange of [^18^F]fluoride to recover [^18^O]H_2_O. The upstream valve selects between incoming fluid from an external addition line (trapping) or the eluent addition position (release). The output of the first stopcock is connected via tubing to a cartridge. The output of the cartridge is connected to a second tube that feeds into a second stopcock valve that selects whether the fluid is directed to a built-in collection vial (trapping) or a line into the position where the reaction vessel is sealed for reagent addition (release). The third stopcock valve is used for cartridge purification of the crude product before transfer to the next reaction vessel. The purification cartridge is installed between the dip tube (for removal of crude product from the reaction vessel) and the tube leading to the stopcock. The outputs of the stopcock are connected to a built-in waste vial (trapping, washing) or an external output line (release) that can be plugged into the next cassette or the high-performance liquid chromatography (HPLC) injection valve. Cartridges can be mounted on clips near the front of the cassettes for convenience.

#### Control system

**Figure 6:**
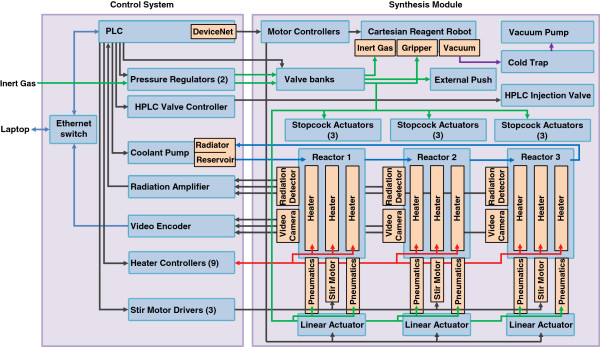
**ELIXYS control system schematic.** Schematic of control components, actuators, and sensors in the control system and synthesis module.

Supporting electronics, pneumatics, and cooling system are enclosed in a separate control system (Figure 6). The system is controlled by a Linux server, which communicates with a programmable logic controller (PLC; CJ2M-CPU31, Omron, Kyoto, Japan) over Ethernet, which in turn drives most of the subsystems including linear actuators, pneumatics, cooling, heating, stirring, and HPLC injection, in addition to reading radioactivity detectors affixed to each reactor. The PLC accomplishes this through several expansion modules (CJ1W-DRM21, CJ1W-AD081-V1, CJ1W-ID261, CJ1W-DA08V, CJ1W-OD261, CJ1W-TC001, Omron).

Five motor controllers are connected to a RoboNET network controller gateway unit (RGW-DV, IAI America Inc., Torrance, CA, USA) in the synthesis module, which is in turn controlled by the PLC. Two of these are pulse motor controllers (RPCON-42P, IAI America Inc.) that drive the *x*- and *y*-axes of the reagent and gas handling robot, a 350- and 100-mm-stroke two-axis linear servomotor (RCP2-SS7R-I-42P-12-350-P1-007L-ML-SP, RCP3-TA7R-I-42P-6-100-P1-N-ML, IAI America Inc.). The other three controllers are linear servomotor controllers (RACON-5, IAI America Inc.) driving the linear servomotor (RCP3-SA3R-I-28P-4-200-P1-P-ML, IAI America Inc.) for the *y*-axis motion of each reactor.

A source of inert gas is dynamically regulated in software from >60 psig down to two different pressures by two analog pressure regulators (ITV1030-31N2L4-Q, SMC Corporation). One pressure line drives the pneumatic actuators (typically set to 60 psig), and the other pressure line drives gas flow for liquid transfers and evaporation (typically 3 to 15 psig). The two lines are distributed to actuators and the gas supplier through solenoid valve banks located in the synthesis module. The higher pressure line is used to raise and lower the reaction vessels using pneumatic cylinders (NCDGBN20-0300, SMC Corporation), turn the stopcock valves via the rotary pneumatic actuators (CRB2BW20-180S, SMC Corporation), raise and lower the two *z*-axis actuators (MXS8-50, SMC Corporation) for the vial gripper and gas supplier, and open and close the vial gripper (MHS2-16D, SMC Corporation). The lower pressure line feeds into the gas supplier that seals to the gas inlet gaskets on top of the cassettes, and an external line that can be used to transfer [^18^F]fluoride from a source vial into the anion exchange cartridge on the cassette, for example.

The control system also houses a number of other components: the solid-state relays (G6B-4BNDDC12, Omron) to switch the heaters on and off for reactor temperature control, the cooling system (coolant pump, reservoir, and radiator fans), a video server (VS8401, Vivotek, San Jose, CA, USA) to encode the analog signals from the reactor cameras into video streams available to the Linux server via Ethernet, and an electronically controlled HPLC injection valve (MHP7900-500-1, Rheodyne, Rohnert Park, CA, USA) connected to a separate semi-preparative HPLC system. Loading of the HPLC loop is performed manually but in the future will be performed automatically [22].

### System operations

The ELIXYS performs automated syntheses by completing a sequence of chemistry unit operations (Table 1). The interactions among the ELIXYS subsystems and disposable cassettes to carry out each operation are described in the following subsections.

**Table 1:** List of unit operations available to build a synthesis sequence

**Unit operation**	**Description of function**
Initialize	Initializes hardware
TrapF18	Trap [^18^F]fluoride from cyclotron or preloaded external vial
EluteF18	Elute [^18^F]fluoride with a reagent from the cassette
Add	Add a reagent from any cassette
Evaporate	Evaporate the contents of a reactor
React	Fully seal a reaction vessel for a reaction
Transfer	Transfer solvents and reaction products from one reaction vessel to another, often using purification cartridges in between
TransferToHPLC^a^	Transfers the contents of the reaction vessel to the HPLC injection loop
ExternalAdd	Move a reactor to its add position for externally adding a reagent
Mix	Mix the contents of a reaction vessel
Install	Move a reactor to a position for reaction vessel removal and/or installation
MeasureRadiation^a^	Measures the radiation level of a given reactor

^a^These unit operations are currently under development.

#### Radioisotope handling

For radiochemistry with [^18^F]fluoride, a preconditioned quaternary methylammonium (QMA) cartridge is installed with Luer fittings between two tubes coming from the cassette, and the source of [^18^F]fluoride (vial or cyclotron) is connected to another tube. (If a vial is used, it is pressurized by an inert gas line controlled by the system.) During trapping, the [^18^F]fluoride source solution flows through the cartridge and [^18^O]H_2_O is collected in a recovery vial. To perform elution, the two stopcock valves are switched and the reagent and gas handling robot drives the eluent from the eluent addition position of the cassette through the cartridge and into the reaction vessel. Multiple elutions or rinses can be performed to increase the efficiency of [^18^F]fluoride collection. PEEK tubing was used for all fluid paths involving [^18^F]fluoride to maximize specific activity [23]. For other radioisotopes, a cartridge may not be necessary and can be bypassed. Radioisotopes may be added to any of the three reactors independently.

#### Reagent handling

To add a particular reagent, the vial gripper moves to the reagent storage position and then lowers itself to a position where it can grasp and lift the vial before moving it to the designated reagent addition location on the specified cassette. To deliver the reagent to the reaction vessel, the gas supplier lowers, the inert gas valve opens, and the vial gripper lowers to place the vial down onto a pair of needles in one of the two reagent addition positions or in the eluent addition position, pressurizing the vial and transferring its contents. The required time for addition of a reagent is generally determined by repeatedly measuring the time needed for complete transfer of the desired liquid and volume at the desired pressure, taking the maximum time value, and multiplying by a safety factor. The entire contents of the reagent vial are delivered at once. After addition is complete, the vial gripper lifts the empty reagent vial, the gas supplier disengages, and the vial is returned to its original storage position. The low-level steps of the Add unit operation are summarized in Additional file 1: Figure S1.

Like all fluidic systems, there are losses associated with dead volumes during liquid transfers. Initial characterization revealed that a small amount (120 ± 20 μL, *n* = 120) of the liquid remains in the reagent vial after addition. To account for this loss, additional reagent can be loaded beforehand into the crimped vials.

#### Reactions

To maintain high internal pressure during superheated reactions, the reaction vessel is sealed by firmly pressing upward against the gasket on the bottom of the cassette. Each cassette has two independent reaction positions to support up to two separate sealed reactions in each reaction vessel (Figure 5). To characterize the seal integrity, approximately 1 mL of anhydrous acetonitrile was sealed and heated at 165°C for 1 h. In all experiments, <14 μL of volume was lost (<1.5%). We believe the actual loss of vapor to be even lower because often some small drops of condensed solvent were observed on the gasket where the reaction vessel had been sealed.

Using a hypodermic needle thermocouple (HN-7-K-TEF, J-KEM Scientific, Saint Louis, MO, USA) pierced through the gasket, we have also compared the internal liquid temperature profile of the reaction vessel contents in the ELIXYS to the profile inside the same vessel immersed in a traditional preheated oil bath (Additional file 1: Figure S2). We found the heating rates and internal liquid temperature to be comparable, but the active liquid cooling of the ELIXYS results in a more rapid decrease in temperature after heating.

After reagents are loaded into the reaction vessel, a reaction can be performed by sealing the vessel against a sealing position on the gasket of the cassette (Figures 4 and 5). The reactor is then heated to the desired temperature, with optional stirring. Once the desired elevated temperature is reached in the heating jacket, heating and stirring are continued for the desired reaction time. After this time elapses, the heaters are turned off and the cooling pump is activated until the heating jacket reaches the desired reduced temperature. Additional cooling of the reaction vessel is necessary to ensure the internal liquid temperature is sufficiently lowered; the desired additional time for cooling can be programmed in the software.

#### Evaporations

Evaporation of solvents occurs by sealing the reaction vessel against the gasket of the cassette at the evaporate position (Figures 4 and 5). The vessel is heated with the option of stirring, and the gas supplier provides both vacuum (to remove vapor) and inert gas (to assist with vapor removal) through the ports on the cassette. The required time for evaporation is generally determined by measuring the maximum time needed for complete evaporation of the solvent from the desired mixture and multiplying by a safety factor. After the desired evaporation time, the reactor is cooled. The low-level steps of the Evaporate unit operation are summarized in Additional file 1: Figure S3.

#### Transfer and purification

Sep-Pak purification cartridges (Sep-Pak), e.g., silica, C18, etc., are connected to designated Luer fittings on the cassette. A dip tube (e.g., made of Teflon® tubing with an outside diameter (OD) of 1/8 in.) is built into the cassette to act as the fluid path for the transfer of crude products. Tubing with an OD of 1/8 in. is necessary when transferring synthesis products that produce precipitates, but 1/16 in. tubing may be installed for smaller volume transfers. The transfer unit operation begins with the reaction vessel sealing against the transfer position on the cassette (Figures 4 and 5). The gas supplier provides inert gas to pressurize the reaction vessel. This moves the fluid through the dip tube and to the Sep-Pak. After the Sep-Pak, a dedicated stopcock valve in the cassette switches between a fluid path towards a waste collection vial installed on the cassette and a tube that can be plumbed to the input of the next cassette. Often, the first step is to trap the crude product onto the Sep-Pak and allow the residual solution to collect in the waste container. The stopcock position is switched, and elution of the desired product into the next reaction vessel is then performed by adding the elution solvent to the first reaction vessel and repeating the transfer unit operation to elute the product from the Sep-Pak.

### Radiosynthesis

#### Materials

No-carrier-added [^18^F]fluoride was produced by the (p,n) reaction of [^18^O]H_2_O (98% isotopic purity, Medical Isotopes, Pelham, NH, USA) in a RDS-112 cyclotron (Siemens, Knoxville, TN, USA) at 11 MeV using a 1-mL tantalum target with Havar foil. 2-*O*-(Trifluoromethylsulfonyl)-1,3,5-tri-*O*-benzoyl-alpha-d-ribo-furanose, 2-*O*-(trifluoromethylsulfonyl)-1,3,5-tri-*O*-benzoyl-alpha-l-ribo-furanose, bis(trimethylsilyl)cytosine, and 5-methyl-2,4-bis[(trimethylsilyl)oxy]pyrimidine were purchased from ABX (Radeberg, Germany). Ethanol (200 proof) was purchased from the UCLA Chemistry Department (Los Angeles, CA, USA). Hydrochloric acid (1 N) was purchased from Fisher Scientific (Pittsburg, PA, USA). Anhydrous grade solvents and all other reagents were purchased from Sigma-Aldrich (Milwaukee, WI, USA). All reagents were used as received. QMA (WAT023525) and silica cartridges (WAT020520 and WAT043400) were purchased from Waters (Milford, MA, USA). The QMA cartridge was preconditioned with 10 mL of 1 M potassium bicarbonate followed by 10 mL of 0.1-μm-filtered 18 MΩ water, and the silica cartridges were preconditioned with 10 mL of anhydrous hexane.

#### Chromatography

Semi-preparative HPLC was performed with a WellChrom K-501 HPLC pump (Knauer, Berlin, Germany), reversed-phase Gemini-NX column (5 μm, 10 × 250 mm, Phenomenex, Torrance, CA, USA), UV detector (254 nm, WellChrom Spectro-Photometer K-2501, Knauer), and gamma radiation detector and counter (B-FC-3300 and B-FC-1000; Bioscan Inc., Washington, DC, USA). The mobile phase for d-[^18^F]FAC was 1% ethanol in 10 mM ammonium phosphate monobasic (flow rate 5 mL/min; retention time 15 min) and for l-[^18^F]FMAU was 4% acetonitrile in 50 mM ammonium acetate (flow rate 5 mL/min; retention time 20 min). Analytical HPLC was performed on a Knauer Smartline HPLC system with a Phenomenex reversed-phase Luna column (5 μm, 4.6 × 250 mm) with in-line Knauer UV (254 nm) and gamma radiation coincidence detector and counter (B-FC-4100 and B-FC-1000). The analytical HPLC mobile phase for d-[^18^F]FAC was 10% ethanol in 50 mM ammonium acetate (flow rate 1 mL/min; retention time 4 min) and for l-[^18^F]FMAU was 10% acetonitrile in 50 mM ammonium acetate (flow rate 1 mL/min; retention time 7 min). All chromatograms were collected using a GinaStar analog-to-digital converter (raytest USA, Inc., Wilmington, NC, USA) and GinaStar software (raytest USA, Inc.) running on a PC.

#### Synthesis protocol

**Figure 7:**
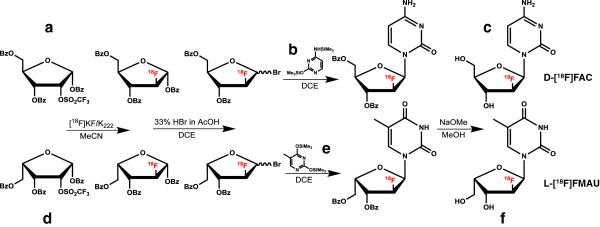
**Reaction mechanisms of d-[**^**18 **^**F]FAC and l-[**^**18**^**F]FMAU.** Syntheses of d-[^18^F]FAC (top) and l-[^18^F]FMAU (bottom). Synthesis protocol of the two tracers differs only in the ribose sugar **(a vs. d)** and base coupling **(b vs. e)** precursors. During HPLC purification, only the beta form (shown here) are collected as final products **(c and f)**.

Synthesis protocols for d-[^18^F]FAC and l-[^18^F]FMAU (Figure 7) were nearly identical, differing only in precursors and HPLC mobile phases, and were programmed using the ELIXYS drag-and-drop software interface [20]. The protocols are a minor adaptation from the literature [17,18]. A summary of the reagents and unit operations used to synthesize the tracers can be found in Additional file 1 (Additional file 1: Tables S1 and S2). Upon completion of each synthesis, the crude product was purified by semi-preparative HPLC and the desired product (beta form, structures c and f in Figure 7) was collected and a sample taken for verification and specific activity analysis by analytical HPLC.

#### In vivo imaging

**Figure 8:**
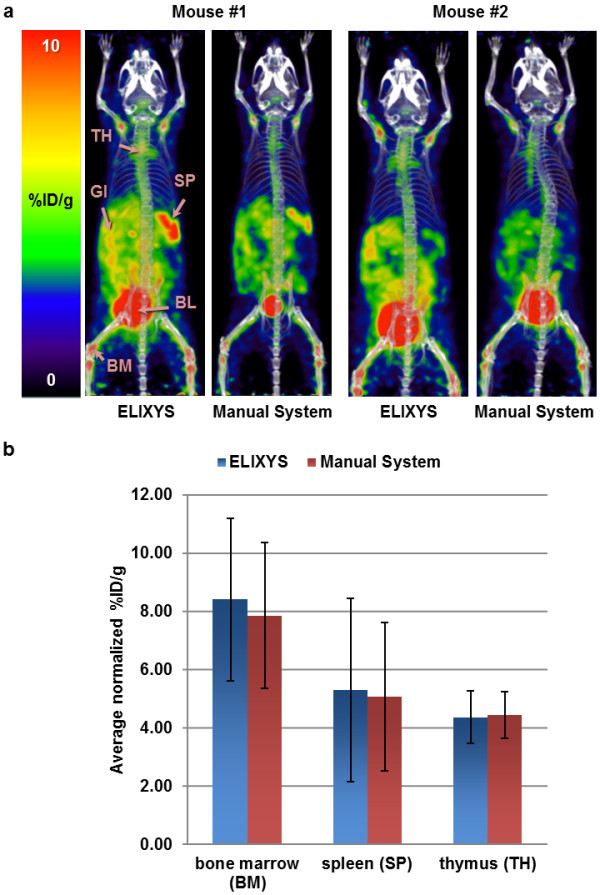
***In vivo *imaging of d-[**^**18**^**F]FAC. (a)** PET/CT imaging of two of the four C57BL/6 mice using d-[^18^F]FAC produced from either ELIXYS or a manually operated apparatus (TH, thymus; GI, gastrointestinal tract; SP, spleen; BL, bladder; BM, bone marrow). **(b)** Percent injected dose per gram (% ID/g) was normalized to muscle tissue and averaged for the four mice in each organ of interest.

Four conscious C57BL/6 mice were injected at the UCLA Ahmanson Translational Imaging Division with 0.74 MBq (20 μCi) of d-[^18^F]FAC (tail vein, 60-min uptake) produced on the ELIXYS. Two days afterward, the same four mice were injected with d-[^18^F]FAC produced at the UCLA Biomedical Cyclotron Facility using a manually operated apparatus [17]. Before scanning, mice were anesthetized with 2% isoflurane and placed in a dedicated imaging chamber designed for use with both the PET and CT systems. Ten-minute whole-body PET images were acquired using a GENISYS^4^ (Sofie Biosciences Inc., Culver City, CA, USA) and then transferred to the micro-CT (ImTek Inc., Knoxville, TN, USA) for an 8-min scan. Parameters for CT acquisition were 70 kVp, 500 μA, and an exposure time of 480 s. A Feldkamp reconstruction algorithm was applied. PET/CT images were fused and analyzed using OsiriX Imaging software (Pixmeo, Geneva, Switzerland). Uptake in the bone marrow (femur), spleen, and thymus were normalized to muscle tissue and respectively averaged over the four mice. Imaging data are presented in Figure 8.

All animal experiments were approved by the UCLA Animal Research Committee and were performed according to the guidelines of the Division of Laboratory Animal Medicine at UCLA.

## Results and discussion

To validate the functionality of the synthesizer, the three-reactor syntheses of d-[^18^F]FAC and l-[^18^F]FMAU were performed. The maximum radioactivity used in the syntheses was approximately 35 GBq. Decay-corrected radiochemical yield, duration of synthesis, and specific activity are listed in Table 2. Synthesis times and yields are comparable to or better than other reports found in the literature [11,16,17,24].

**Table 2:** Comparison of synthesis data

**Radiotracer**	**Reference**	**Radiochemical yield (%)**	**Duration of synthesis (min)**	**Specific activity (GBq/μmol)**
[^18^F]FAC	Amarasekera et al. [17]	39 ± 5 (*n* = 13)	~240	>37
	This work	31 ± 5 (*n* = 6)	165	37 to 44
[^18^F]FMAU	Alauddin et al. [11]	20 to 30	210 to 240^a^	85.1
	Mangner et al. [24]	42.1 ± 12.1 (*n* = 9)	160	111^b^
	Li et al. [16]	12 ± 3 (*n* = 4)	150^a^	14.2 ± 1.2
	Amarasekera et al. [17]	35 ± 6 (*n* = 10)	~240	>37
	This work	46 ± 1 (*n* = 6)	170	100 to 170

Yield, synthesis duration, and specific activity of two tracers produced with the ELIXYS radiosynthesizer compared to values found in literature. ^a^Endpoint not specified, but presumably includes HPLC purification; ^b^time point not specified, but presumably at time of injection.

The ELIXYS currently supports PET tracer production in our preclinical facility, and therefore, products were confirmed by coinjection with cold standard into the analytical HPLC, and radiochemical purity was found to be >99% for both tracers. However, numerous batches of d-[^18^F]FAC (as well as other tracers) have been subjected to the complete set of quality assurance tests required for clinical use and have passed. These tests include residual solvent analysis via gas chromatography, Kryptofix K_222_ spot test, filter integrity test, radionuclide identity by half-life and energy, visual inspection of optical clarity, as well as pyrogenicity and sterility. We are currently updating the software for compliance with current good manufacturing practice, and the updated system will be placed in a clinical facility for production of tracers under 21 CFR 212 regulations or USP 823 guidelines.

*In vivo* imaging using d-[^18^F]FAC produced on ELIXYS and that produced by the UCLA Biomedical Cyclotron Facility in a manually operated apparatus [17] showed comparable images and uptake as expected in the gastrointestinal tract (GI) and hematopoietic organs (Figure 8) [25]. Though there appears to be higher ‘noise’ (muscle uptake) in the images of mice injected with ELIXYS-produced d-[^18^F]FAC, there is also higher ‘signal’ (organ uptake). Indeed, whole-body regions of interest excluding the tail showed that the total amount of activity was higher in the images of the mice injected with the ELIXYS-produced tracer. We suspect that there may have been variations in the injections, e.g., more tracer left in the tail in some of the images, leading to a lower amount of activity in circulation. To remove these biases, organ uptake was normalized to a region of muscle tissue that showed no significant uptake.

The disposable cassette approach has allowed for multiple tracers in addition to d-[^18^F]FAC and l-[^18^F]FMAU to be readily synthesized, including 2-deoxy-2-[^18^F]fluoro-5-ethyl-β-d-arabinofuranosyluracil (d-[^18^F]FEAU) [26] as well as 2-[^18^F]fluoro-2-deoxy-d-glucose ([^18^F]FDG), 3-deoxy-3-[^18^F]fluoro-l-thymidine ([^18^F]FLT), [^18^F]fallypride, 9-(4-[^18^F]-fluoro-3-hydroxymethylbutyl)-guanine ([^18^F]FHBG), and *N*-succinimidyl-4-[^18^F]fluorobenzoate ([^18^F]SFB) (results to be published separately), by simply switching cassettes and software programs. No hardware or plumbing changes were needed between productions of different tracers.

## Conclusion

We have developed a new, versatile radiosynthesizer that is suitable both for reaction development and routine production of PET tracers. The ELIXYS synthesizer contains very few wetted fittings and tubing compared to other radiosynthesizers, yet through the unique use of motion to implement dynamically reconfigurable fluid paths, it is capable of diverse syntheses without hardware customization. It has been designed to be capable of diverse syntheses requiring up to three reaction vessels, high-pressure and high-temperature reactions, as well as sensitive, corrosive, and volatile reagents.

To validate this new system, the three-reactor syntheses of d-[^18^F]FAC and l-[^18^F]FMAU were demonstrated, and yields and synthesis times were found to be comparable to other reports. Several additional tracers of varying complexity have also been successfully synthesized on this system.

## Competing interests

RMvD and MEP own equity in Sofie Biosciences, Inc. GJS and MDM are employees of Sofie Biosciences, Inc. SBC and KMQ have worked for Sofie Biosciences, Inc. as paid consultants. ML, JC, HEH, and BM declare that they have no competing interests.

## Authors’ contributions

ML, KMQ, SBC, GJS, MDM, and RMvD designed the synthesizer. ML, KMQ, SBC, and BM built and tested the synthesizer. HEH contributed key design elements to the synthesizer and software. ML, KMQ, SBC, MDM, and RMvD designed the software architecture and user interface. KMQ and SBC implemented and tested the software. ML, KMQ, and SBC designed and performed experiments to characterize the synthesizer. ML performed the radiosyntheses and analyses of synthesis data. JC helped perform the radiosyntheses and collect specific activity data. MEP, MDM, and RMvD conceived the project. ML and RMvD wrote the manuscript. All authors read and approved the final manuscript.

## Supplementary Material

Additional file 1: Figure S1 Flowchart of ‘Add’ unit operation. Sequence of low level steps required to add the contents of a reagent placed in the second reagent position of the first cassette to the first reaction vessel. The user need only choose the ‘Add’ unit operation and set a few parameters; the low level details are carried out automatically. **Figure S2**. Internal temperature of liquid in the reaction vessel during heating and cooling in the ELIXYS system and in an oil bath. **Figure S3**. **(a)** Sequence of low level steps required to evaporate the contents of the first reaction vessel. **(b)** Gas supplier is positioned over the vacuum and inert gas ports of the cassette. **(c)** Gas supplier is lowered, supplying vacuum and inert gas to the reaction vessel. **Table S1**. List of reagents installed into the three cassettes to synthesize D-[^18^F]FAC and L-[^18^F]FMAU. Externally added reagents (HBr and DCE) do not have designated locations on the cassette and can be programmed with the ExternalAdd unit operation. **Table S2**. List of unit operations to synthesize both D-[^18^F]FAC and L-[^18^F]FMAU on the ELIXYS. Click here for file
