# Supplementary material for: ELIXYS - a fully automated, three-reactor high-pressure radiosynthesizer for development and routine production of diverse PET tracers
Source: EJNMMI Res. 2013 Jul 12;3:52. doi: 10.1186/2191-219X-3-52 (PMC3717127; doi:10.1186/2191-219X-3-52)
Supplement: Additional file 1: Figure S1 — Flowchart of ‘Add’ unit operation. Sequence of low level steps required to add the contents of a reagent placed in the second reagent position of the first cassette to the first reaction vessel. The user need only choose the ‘Add’ unit operation and set a few parameters; the low level details are carried out automatically. Figure S2. Internal temperature of liquid in the reaction vessel during heating and cooling in the ELIXYS system and in an oil bath. Figure S3. (a) Sequence of low level steps required to evaporate the contents of the first reaction vessel. (b) Gas supplier is positioned over the vacuum and inert gas ports of the cassette. (c) Gas supplier is lowered, supplying vacuum and inert gas to the reaction vessel. Table S1. List of reagents installed into the three cassettes to synthesize D-[18F]FAC and L-[18F]FMAU. Externally added reagents (HBr and DCE) do not have designated locations on the cassette and can be programmed with the ExternalAdd unit operation. Table S2. List of unit operations to synthesize both D-[18F]FAC and L-[18F]FMAU on the ELIXYS. [file 2191-219X-3-52-S1.pdf]

Supplementary Material for *EJNMMI Research* Manuscript:

Title:
ELIXYS – A fully automated, three-reactor high-pressure radiosynthesizer for development and routine production of diverse PET tracers

Authors:
Mark Lazari[1,2,3], Kevin Quinn[2,3], Shane Claggett[2,3], Jeffrey Collins[2,3], Gaurav J. Shah[2,3,4], Henry Herman[2,3], Brandon Maraglia[2,3,4], Michael E. Phelps[2,3], Melissa Moore[2,3,4], R. Michael van Dam*[,1,2,3]

Affiliations:
[1]Department of Bioengineering, Henry Samueli School of Engineering, UCLA, Los Angeles, CA, 90095, USA;
[2]Department of Molecular & Medical Pharmacology, David Geffen School of Medicine, UCLA, Los Angeles, CA, 90095, USA;
[3]Crump Institute for Molecular Imaging, David Geffen School of Medicine, UCLA, Los Angeles, CA, 90095, USA;
[4]Sofie Biosciences, Inc., Culver City, CA 90230, USA;

*Corresponding Author:
R. Michael van Dam
mvandam@mednet.ucla.edu
4323 CNSI, 570 Westwood Plaza, Building 114
Los Angeles, CA 90095
USA
Phone: +1-310-206-6507
Fax: +1-310-206-8975

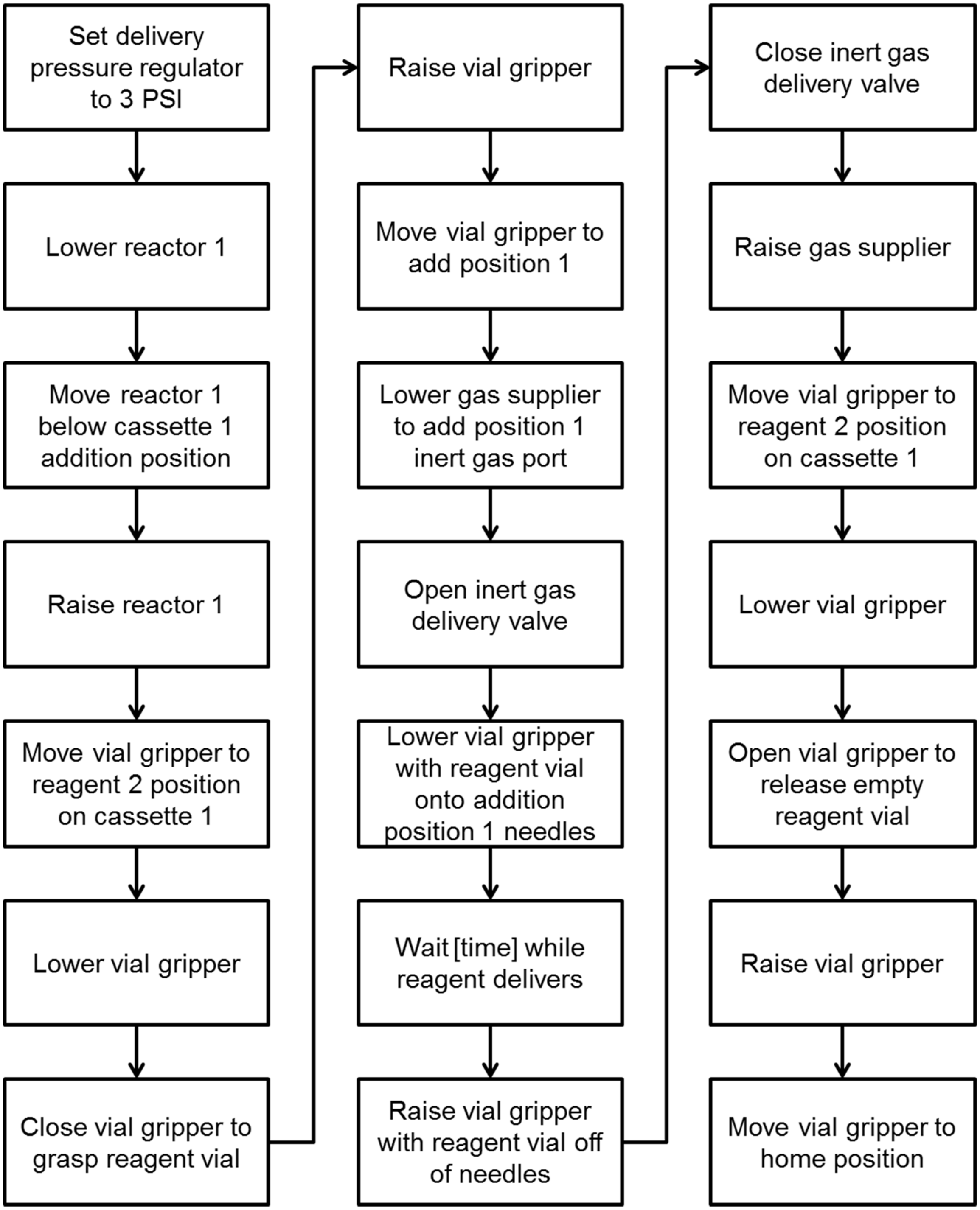

Supplementary Figure 1. Flowchart of 'Add' unit operation. Sequence of low level steps required to add the contents of a reagent placed in the second reagent position of the first cassette to the first reaction vessel. The user need only choose the 'Add' unit operation and set a few parameters; the low level details are carried out automatically.

Internal Liquid Temperature Profile Comparison of ELIXYS and Oil Bath Heating

The comparison of internal liquid temperatures was performed as follows. The reaction vessel was filled with ~1mL of acetonitrile with a hypodermic thermocouple tip submerged in the liquid. For the ELIXYS test, the reactor was heated to 160°C, sealed against a modified cassette with protruding thermocouple and held at temperature for 15 min. For the oil bath test, the reaction vessel was sealed with a silicone stopper pierced with the thermocouple and placed in the preheated 160°C oil bath for 15 min. Active cooling was used for the ELIXYS and passive ambient air cooling was used for the oil bath, as is conventional for most oil bath heated systems. As Supplementary Figure 1 shows, ramping time for heating the solvent is comparable. Both temperatures stabilize at a temperature slightly lower than the set point. This is normal in all radiosynthesizers if the set point is above the solvent boiling point, with the difference depending on the unique thermal characteristics (i.e. heat sources and heat sinks) of the system. Also shown in Supplementary Figure 1, the ELIXYS cools the solvent much faster than simply removing the vessel from the oil bath. If compressed-air cooling is used, the cooling rate is intermediate between these rates (data not shown).

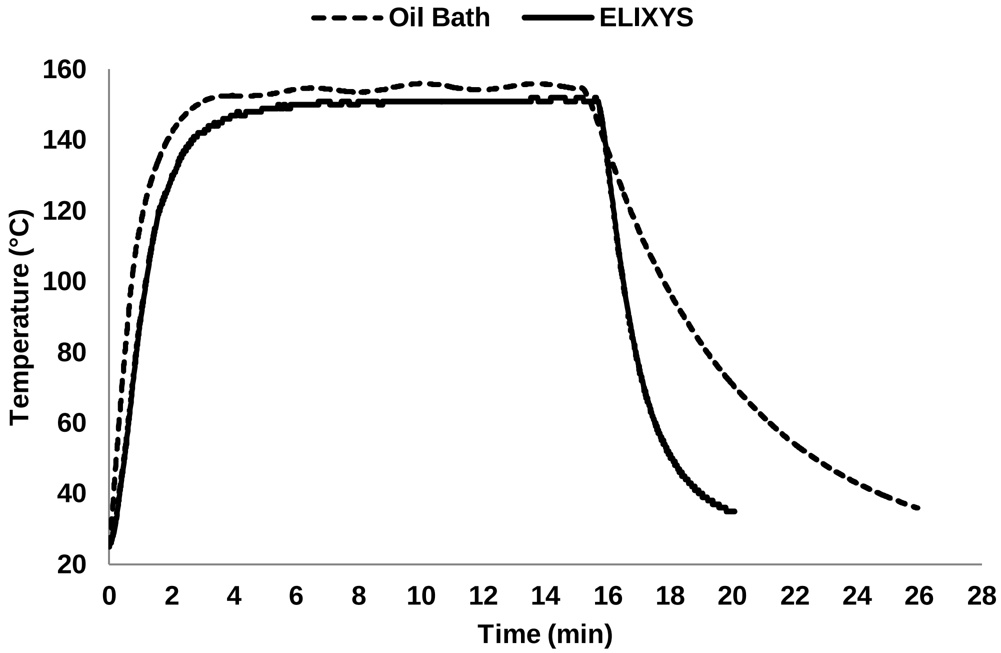

Supplementary Figure 2. Internal temperature of liquid in the reaction vessel during heating and cooling in the ELIXYS system and in an oil bath.

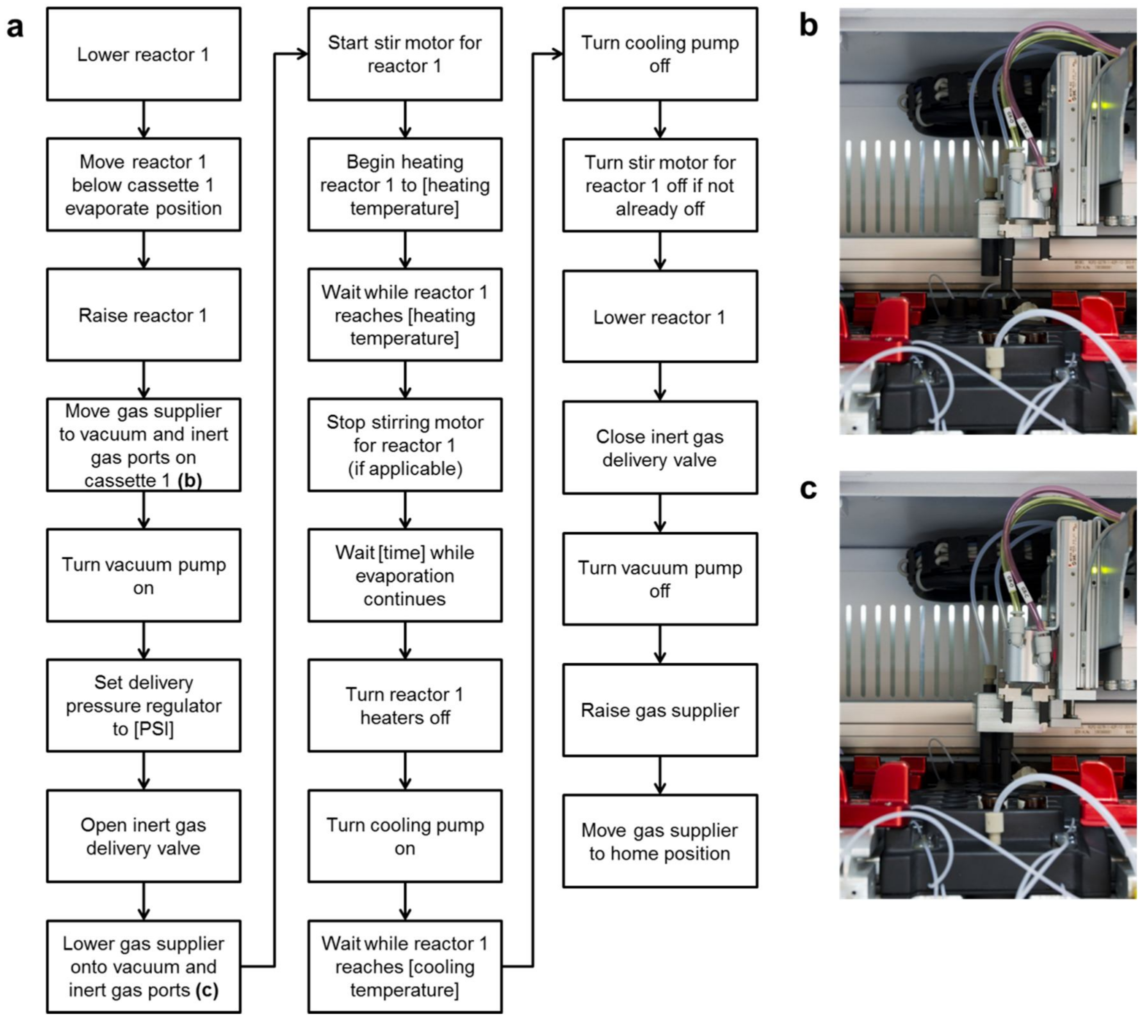

Supplementary Figure 3. (a) Sequence of low level steps required to evaporate the contents of the first reaction vessel. (b) Gas supplier is positioned over the vacuum and inert gas ports of the cassette. (c) Gas supplier is lowered, supplying vacuum and inert gas to the reaction vessel.

Reagent ID[a]	Name[b]	Description
1-2	Eluent	1mg potassium carbonate and 10mg of Kryptofix dissolved in 0.800mL of 3:5 water:acetonitrile
1-3	MeCN-1	1.2mL, anhydrous
1-4	MeCN-2	1.2mL, anhydrous
1-5	Precursor 1	10mg dissolved in 1mL anhydrous acetonitrile
1-6	EtOAc-1	2mL, anhydrous
1-7	EtOAc-2	2mL, anhydrous
2-1	Toluene	0.900mL, anhydrous
2-2	Precursor 2	30mg ([^{18}F]FAC) or 107mg ([^{18}F]FMAU) dissolved in 1mL anhydrous DCE
2-3	DCM:MeOH-1	2mL 9:1 (v/v)
2-4	DCM:MeOH-2	2mL 9:1 (v/v)
2-5	DCM:MeOH-3	2mL 9:1 (v/v)
3-1	NaOMe	0.700mL of 0.5M in methanol
3-2	HCl	0.390mL of 1N
3-3	Water	1mL water
2-ExternalAdd1	HBr	0.150mL, 33% in acetic acid
2-ExternalAdd1	DCE	0.600mL, anhydrous DCE

(a) Notation: Cassette number – reagent position

(b) Name given in the software for referring to the reagents. Abbreviations: MeCN, acetonitrile; Precursor 1, 2-O-(Trifluoromethylsulfonyl)-1,3,5-tri-O-benzoyl-alpha-D-ribofuranose (D-[^{18}F]FAC), 2-O-(Trifluoromethylsulfonyl)-1,3,5-tri-O-benzoyl-alpha-L-ribofuranose (L-[^{18}F]FMAU); EtOAc, ethyl acetate; Precursor 2, bis(trimethylsilyl)cytosine (D-[^{18}F]FAC), 5-methyl-2,4-bis[(trimethylsilyl)oxy]pyrimidine (L-[^{18}F]FMAU); DCM, dichloromethane; MeOH, methanol; NaOMe, sodium methoxide; HCl, hydrochloric acid; HBr, hydrobromic acid; DCE, 1,2-dichloroethane.

Supplementary Table 1. List of reagents installed into the three cassettes to synthesize D-[^{18}F]FAC and L-[^{18}F]FMAU. Externally added reagents (HBr and DCE) do not have designated locations on the cassette and can be programmed with the ExternalAdd unit operation.

#	Unit Operation	Description
1	INITIALIZE	Initializes hardware.
2	TRAPF18	Trap [^{18}F]fluoride for 120s at 3 PSI from external vial.
3	ELUTEF18	Elute [^{18}F]fluoride with Eluent for 120s at 3 PSI.
4	EVAPORATE	Evaporate reactor 1 at 110°C for 360s with 15 PSI and vacuum.
5	ADD	Add MeCN-1 to reactor 1.
6	EVAPORATE	Evaporate reactor 1 at 110°C for 150s with 10 PSI and vacuum.
7	ADD	Add MeCN-2 to reactor 1.
8	EVAPORATE	Evaporate reactor 1 at 110°C for 150s with 10 PSI and vacuum.
9	ADD	Add Precursor 1 to reactor 1.
10	REACT	React reactor 1 in position 1 for 900s at 160°C, cooling at 35°C for 120s.
11	TRANSFER	Trap crude product from reactor 1 onto Silica purification cartridge with 3 PSI for 60s.
12	ADD	Add EtOAc-1 to reactor 1.
13	TRANSFER	Elute product from Silica purification cartridge to reactor 2 with 3 PSI for 30s.
14	EVAPORATE	Evaporate reactor 2 at 80°C for 150s with 10 PSI and vacuum.
15	ADD	Add EtOAc-2 to reactor 1.
16	TRANSFER	Elute product from Silica purification cartridge to reactor 2 with 3 PSI for 30s.
17	EVAPORATE	Evaporate reactor 2 at 80°C for 150s with 10 PSI and vacuum.
18	EXTERNALADD	Add HBr immediately followed by DCE through ExernalAdd-1 of cassette 2.
19	REACT	React reactor 2 in position 1 for 600s at 80°C, cooling to 35°C for 120s.
20	EVAPORATE	Evaporate reactor 2 at 80°C for 150s with 10 PSI and vacuum.
21	ADD	Add Toluene to reactor 2.
22	EVAPORATE	Evaporate reactor 2 at 110°C for 150s with 10 PSI and vacuum.
23	ADD	Add Precursor 2 to reactor 2.
24	REACT	React reactor 2 in position 2 for 1800s at 165°C, cooling to 35°C for 120s.
25	TRANSFER	Trap crude product from reactor 2 onto Silica purification cartridge with 7 PSI for 60s.
26	ADD	Add DCM:MeOH-1 to reactor 2.
27	TRANSFER	Elute product from Silica purification cartridge to reactor 3 with 10 PSI for 30s.

28	EVAPORATE	Evaporate reactor 3 at 80°C for 110s with 10 PSI and vacuum.
29	ADD	Add DCM:MeOH-2 to reactor 2.
30	TRANSFER	Elute product from Silica purification cartridge to reactor 3 with 10 PSI for 30s.
31	EVAPORATE	Evaporate reactor 3 at 80°C for 110s with 10 PSI and vacuum.
32	ADD	Add DCM:MeOH-3 to reactor 2.
33	TRANSFER	Elute product from Silica purification cartridge to reactor 3 with 10 PSI for 30s.
34	EVAPORATE	Evaporate reactor 3 at 80°C for 120s with 10 PSI and vacuum.
35	ADD	Add NaOMe to reactor 3.
36	REACT	React reactor 3 in position 1 for 300s at 105°C, cooling to 35°C for 120s.
37	ADD	Add HCl to reactor 3.
38	MIX	Mix the contents of reactor 3 for 20s.
39	EVAPORATE	Evaporate reactor 3 at 80°C for 120s with 10 PSI and vacuum.
40	ADD	Add Water to reactor 3.
41	MIX	Mix the contents of reactor 3 for 20s.
42	TRANSFERTOHPLC	Transfer contents of reactor 3 to the HPLC injection loop.

Supplementary Table 2. List of unit operations to synthesize both D-[^{18}F]FAC and L-[^{18}F]FMAU on the ELIXYS.
